# Demand generation and social mobilisation for integrated community case management (iCCM) and child health: Lessons learned from successful programmes in Niger and Mozambique

**DOI:** 10.7189/jogh.04.020410

**Published:** 2014-12

**Authors:** Alyssa B Sharkey, Sandrine Martin, Teresa Cerveau, Erica Wetzler, Rocio Berzal

**Affiliations:** 1 UNICEF, New York, NY, USA; 2 Malaria Consortium, Maputo, Mozamique; 3 Save the Children, Maputo, Mozamique; 4 UNICEF, Niamey, Niger

## Abstract

**Aim:**

We present the approaches used in and outcomes resulting from integrated community case management (iCCM) programmes in Niger and Mozambique with a strong focus on demand generation and social mobilisation.

**Methods:**

We use a case study approach to describe the programme and contextual elements of the Niger and Mozambique programmes.

**Results:**

Awareness and utilisation of iCCM services and key family practices increased following the implementation of the Niger and Mozambique iCCM and child survival programmes, as did care–seeking within 24 hours and care–seeking from appropriate, trained providers in Mozambique. These approaches incorporated interpersonal communication activities and community empowerment/participation for collective change, partnerships and networks among key stakeholder groups within communities, media campaigns and advocacy efforts with local and national leaders.

**Conclusions:**

iCCM programmes that train and equip community health workers and successfully engage and empower community members to adopt new behaviours, have appropriate expectations and to trust community health workers’ ability to assess and treat illnesses can lead to improved care–seeking and utilisation, and community ownership for iCCM.

The success of integrated community case management (iCCM) programmes to treat childhood illnesses requires attention to appropriate supply elements (including trained community health workers and adequate commodities) as well as demand elements that promote timely and appropriate care–seeking and treatment utilization [1–3]. The factors that influence demand for child health services are multiple and include financial barriers, non–financial barriers (such as geographic access, caregiver understanding of the illness, preferences for home management and alternative treatments, and limited decision–making autonomy to seek care), as well as caregiver perspectives on the quality of services provided [4–10]. When attention is paid to dismantling these barriers, providing acceptable services and implementing strategies to mobilize and empower families and communities, care–seeking and treatment utilisation can be impacted in a positive way [6,11,12].

Published studies shed some light on the relationship between iCCM services and demand generation. For example, studies from Cameroon [13], Zambia [14,15] and Uganda [16] have reported increased levels of care–seeking for, and utilisation of, appropriate treatment of childhood illnesses where iCCM has been implemented compared to areas without these services. Concurrent reductions in the use of home care as a first treatment [13–15], public facilities [14,15] and ‘other’ services (including traditional healers) [14] as sources of care and treatment for childhood illnesses have been reported in sub–Saharan African settings as well. In addition, two studies from Uganda have reported improvements in the timeliness of care–seeking and treatment uptake in iCCM programme areas [16,17]. However, in Ethiopia, where an ambitious iCCM scale–up is currently being implemented, a recent study also noted that having community–based services in place was not enough to drive appropriate uptake by local populations, and that demand creation activities to promote use of services must be a key element [18].

In this paper, we present case studies of iCCM programmes in Niger and Mozambique that included a specific focus on demand generation and social mobilisation related–efforts. The Niger experience is based on a comprehensive approach incorporating behavioural change communication, social mobilisation and advocacy for eight key family practices. The Mozambique experience is based on a comprehensive approach combining community engagement strategies with efforts to ensure effective access to trained and equipped community health workers, particularly in areas with high levels of unmet need. Our hypothesis is that holistic iCCM programmes that not only address supply–side determinants of coverage, but also are responsive to demand–side elements at inception and with appropriate community engagement, can improve care–seeking and utilisation among families with sick children. The case studies show how this hypothesis was tested in two diverse settings.

## NIGER EXPERIENCE: BEHAVIOUR AND SOCIAL CHANGE FOR KEY FAMILY PRACTICES

### Setting

With a population of 17.1 million people in 2012, Niger has one of the lowest Human Development Index rankings in the world [19] and its under–5 mortality rate of 114 is one of the world’s highest [20]. However, Niger has also made significant reductions in under–5 mortality since 1990, and is amongst 23 of the 74 Countdown countries on track to achieve its millennium development goal for child mortality (MDG4) [21]. Key factors leading to this decline were the establishment of health posts (peripheral structures in villages with at least 5000 people and located more than 10 km from health centres) in 2000 and the establishment of free health care for pregnant women and children under 5 which began in 2006 [20]. Beginning in 2008, paid community health workers in public health posts were authorised to provide iCCM for children with fever or malaria, suspected pneumonia, and diarrhoea [20]. However, even following these advancements, challenges and barriers to effective care–seeking and treatment utilisation remained in Niger, and the overall level of child mortality continued to be unacceptably high.

### Overview of the approach

Between 2006 and 2008, the government of Niger in collaboration with UNICEF and other non–governmental and media partners began planning for a model programme of communication for social and behaviour change for child survival. At the national level, a multisectoral team was identified with representatives of the ministry of health, the ministry of communication, the ministry of water resources, and the ministry of education and decentralisation. Together the team identified the key family practices (‘*les pratiques familiales essentielles’* or ‘*PFEs*’) critical to improve child survival: exclusive breastfeeding, sleeping under insecticide–treated bednets, oral rehydration solution (ORS) for treatment of diarrhoea, hand washing with soap, complementary feeding, use of preventive health services like vaccinations and treatment for childhood illnesses. (Promotion of birth spacing started in 2012 as the eighth key family practice (KFP).) In addition, the team identified a three pronged approach to promote social and behavioural change including: social and behaviour change communication, social mobilisation and advocacy efforts (**Box 1**). Together these partners worked to secure the necessary financial and human resources to test the approach, as well as to begin to establish coordination mechanisms at both national and local levels.

Box 1 **Niger’s three pronged approach to promote social and behavioural change for child survival**

**1. Social and behaviour change communication**

• Interpersonal communication (IPC) using relais communautaires and participatory communication mechanisms such as local and traditional media, cinema, theatre and community radio

• Community empowerment/participation for collective change through community learning activities, community–led design, and implementation and monitoring of action plans

**2. Social mobilisation**

• Creation of partnerships and networks (traditional chiefs, women and youth)

• Media campaigns and proximity media (cinema forum, community radios, theatre)

**3. Advocacy**

• Local (imams, traditional chiefs) and national partners

Tools utilised within the Niger programme are available at: http://ccmcentral.com/iccm-symposium/tools/#tab14

In 2008, the Maradi and Zinder regions were identified as the setting in which to test the approach. Within these regions, five communes (with 50 intervention villages and 25 comparison villages) were identified [22].

With the support of UNICEF, local government teams and non–governmental staff convened advocacy meetings in each village to introduce the approach and build local trust. In addition, community workers were elected (a two–day process based on defined criteria) and trained over a period of seven days. These individuals were then charged with leading implementation of the approach and guiding activities locally.

**Figure 1:**
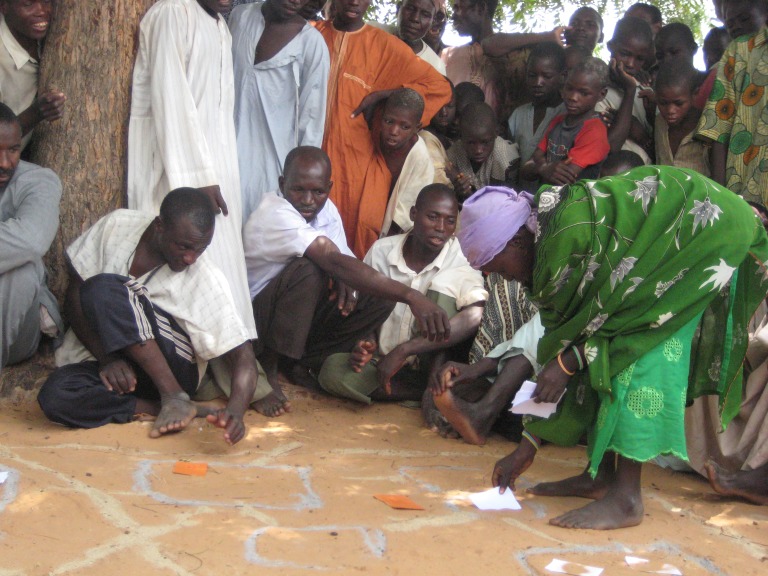
A participatory community assessment in Niger. Photo credit: UNICEF NIGER.

Next, local core groups were created to promote the key family practices. In order to ensure that the approach was largely community–based and community–driven, *relais communautaires* (a cadre of community volunteers) were engaged along with key community leaders such as imams and traditional leaders. Together these partners conducted participatory community and household assessments to identify the main drivers of local behaviours and analyse barriers to high coverage of each of the eight family practices. Open dialogue is encouraged during these assessments, in order to ensure that both constraints and locally acceptable solutions are discussed, and that consensus regarding the need for change can be reached. Based on the findings of these assessments, the partners work together to develop village ‘Plans of Action’ (**Figure 1**).

Next, partnerships and networks were created with local media, mobile cinema vans, theatre, and community radio activities which encouraged local participation in ‘debates’ around key issues. In addition, *relais* organized lectures and home visits around the family practices, traditional chiefs were engaged to promote daily hygiene, and imams were engaged to promote breastfeeding before evening prayers. Mothers’ and youths’ peer–support groups were also convened.

Volunteers and local leaders monitored progress with tools created to assess household adoption of the key family practices and monthly village meetings were held to discuss progress. The information from this monitoring was also shared with health workers based at health centres. Annual village meetings were held to assess progress and revise action plans as needed. The core teams created annual celebrations of village and family ‘champions,’ established individuals as ‘agents of change,’ and identified ‘model villages’ when at least 70 percent of families had adopted three or more of the key family practices. ‘Model mothers’ in these areas are awarded with soap, mosquito nets or a radio.

### Scaling up the approach

By 2011, the government of Niger and UNICEF had worked together to extend the approach from five to 30 communes, representing a total population of more than 1 million people. As it was expanded, the approach was integrated into Commune Development Plans. Multisectoral and synergistic activities were incorporated into the approach in some communes based on other programmatic opportunities relating to building external partnerships (for example with the World Food Programme, World Bank or the Food and Agriculture Organisation) or intersectoral programmes. Examples included a cash transfer project, community led total sanitation (CLTS) activities, and the development of ‘education for health’ curricula for children.

### Data used and programme results

Several data sources were used to assess the approach. In 2011, a KFP survey was conducted in the 176 villages in which the intervention was implemented and in 25 control areas [22]. The objectives of this study were to 1) compare coverage of the KFPs with similar areas that were not exposed to the intervention, 2) provide information on the effectiveness of various strategies used in communication for social change and behavior, and 3) collect baseline data for a separate pilot project on social nets. In addition, between July 2011 and December 2012, a qualitative study was conducted in two villages in Maradi, two in Tahoua and one in Zinder to assess perceptions of KFPs and their related practices [23]. This qualitative study also assessed families’ constraints and obstacles to the adoption of the KFPs, as well as the perceptions of the local population of the implementation of the program, including the role of key players such as project staff, health workers, community liaisons, and community leaders. In each setting, the research team met with local stakeholders to discuss the research plans and objectives, and later the preliminary research findings to obtain inputs and feedback. Finally, in 2012, UNICEF supported additional data collection within the national Demographic and Health Survey in order to ensure availability of disaggregated data for the intervention areas and to enable comparisons with the national situation [24]. This survey examined a representative sample of 5875 households consisting of twelve departments in the intervention areas and provided information on coverage of the key family practices.

**Table 1** shows some of the differences in uptake of some key family practices in the intervention areas and nationally in 2012.

**Table 1:** Uptake of some key family practices in Niger intervention and comparison areas, 2012 (percent and 95% confidence intervals, unweighted data)*

Key family practice	Intervention areas	Comparison areas
Children ages 0–5 y with diarrhoea in the previous 2 weeks who received treatment with oral rehydration salts (ORS)	58.3 (51.4–65.1)	29.1 (20.6–37.6)
Children ages 0–5 y who were ill with fever in the previous 2 weeks and who received Artemisinin–based Combination Therapy (ACT) for treatment of malaria	5.3 (3.0–7.5)	0.4 (0.0–1.3)
Mother or caregivers who reported that their children ages 0–5 y slept under an insecticide–treated bednet the previous night	98.6 (97.3–99.3)	99.4 (97.4–99.9)
Children ages 12–23 mo who receiving complete vaccinations	47.8 (43.7–52.0)	38.5 (33.1–44.4)
Early breastfeeding initiation (within 1 h of delivery)	88.7 (85.9–91.5)	76.5 (71.2–81.7)
Exclusive breastfeeding (0 to 6 mo)	77.4 (73.6–81.1)	53.7 (47.1–60.3)
Complementary feeding (6 to 23 mo)	44.1 (40.5–47.6)	26.2 (21.6–30.8)
Knowledge of danger signs of diarrhoea, pneumonia and malaria	30.1 (26.8–33.4)	8.8 (5.9–11.8)

*Source: Institut National De La Statistique – Niger (2012) Enquete Quantitative Relative ŕ la Recherche Action sur les Pratiques Familiales Essentielles, La Nutrition et Les Dépenses de Consommation des Ménages dans les Régions de Maradi, Zinder, Tahoua et Tillaberi: Rapport d’analyse. Niamey: Institut National De La Statistique – Niger.

**Figure 2:**
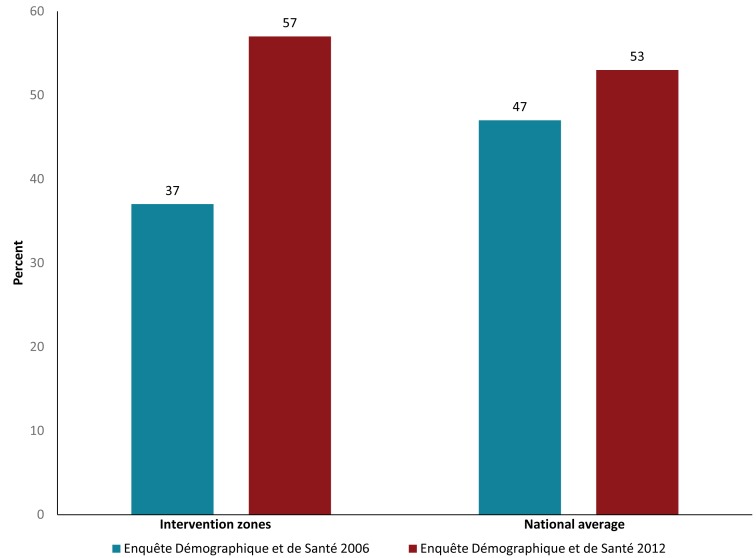
Percent of children with symptoms of acuter respiratory infections (ARI) for whom care was sought, intervention zones and national average, Niger 2006 to 2013. Sources: Institut National de la Statistique (INS) [Niger], Unicef et ICF International 2012. *Enquęte Démographique et de Santé dans les Zones d’Intervention du Programme de Coopération de l’UNICEF au Niger, 2012*. Rockville, Maryland, USA: INS, Unicef et ICF International; and Institut National de la Statistique (INS) et Macro International Inc. Enquęte Démographique et de Santé et à Indicateurs Multiples du Niger 2006. Calverton, Maryland, USA: INS et Macro International Inc.; Février 2007. http://dhsprogram.com/pubs/pdf/FR193/FR193-NI06.pdf.

Data from the 2006 and 2012 Enquęte Démographique et de Santé et ŕ Indicateurs Multiples (EDSN–MICS) surveys indicate that, with the exception of antimalarial treatment following the change in policy to provide Artemisinin–based Combination Therapy (ACT) and use of rapid diagnostic tests (RDTs), there were improvements in several key family practices over time nationally (**Table 2**). These improvements were even more pronounced in the intervention areas. For example, **Figure 2** shows changes in the percent of children with symptoms of acute respiratory infections (ARI) for whom care was sought in the intervention zones vs the national average between 2006 and 2012.

**Table 2:** Uptake of key family practices relating to pneumonia, diarrhoea and malaria in Niger, 2006 and 2012 (percent and 95% confidence intervals)*

Key family practice	Niger, 2006	Niger, 2012
Children ages 0–5 y with diarrhoea in the previous 2 weeks who received treatment with oral rehydration salts	17.6 (15.3–20.0)	44.3 (41.1–47.5)
Children ages 0–5 y with suspected pneumonia in the previous 2 weeks who were taken to an appropriate health–care provider†	46.5 (40.9–52.1)	53.1 (46.5–59.6)
Children ages 0–5 y who were ill with fever in the previous 2 weeks and who received any antimalarial medicine	33.0 (29.6–36.7)	19.2 (16.7–22.0)
Children ages 0–5 y who slept under an insecticide treated mosquito net the previous night	7.2 (6.1–8.4)	20.7 (18.5–23.0)
Children who received complete vaccinations (12–23 mo)	29.0 (25.5–32.5)	52.0 (48.6–55.3)

*Source: Institut National de la Statistique (INS) et Macro International Inc. 2007. *Enquęte Démographique et de Santé et ŕ Indicateurs Multiples du Niger 2006*. [Data set]. NIKR51FL.DTA. Calverton, Maryland, USA: INS et Macro International Inc.; Institut National de la Statistique (INS) [Niger], Unicef et ICF International 2012. *Enquęte Démographique et de Santé dans les Zones d’Intervention du Programme de Coopération de l’Unicef au Niger, 2012*. [Data set]. NIKR61FL.DTA. Rockville, Maryland, USA: INS, Unicef et ICF International.

†Appropriate health–care provider refers to all public or private facilities, except for pharmacies and shops, traditional healers and other non–medical facilities.

**Figure 3:**
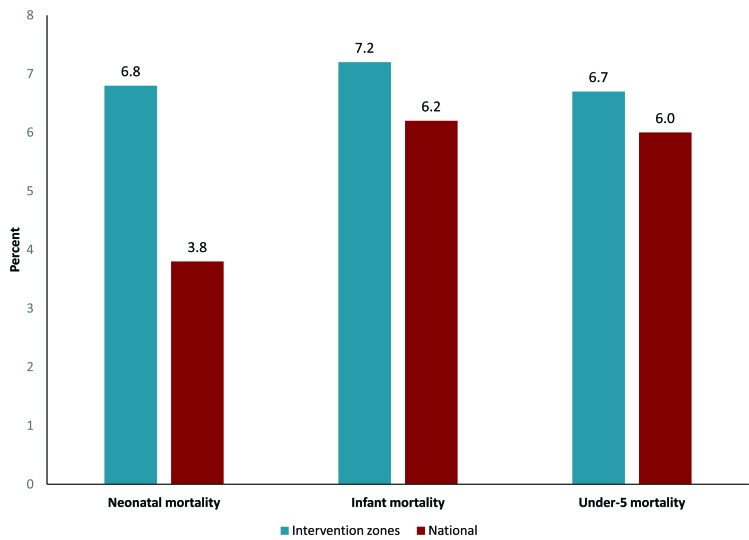
Average annual rate of reduction in child mortality (percent) in the intervention zones compared to the national rate, Niger 2006–2012. Sources: Institut National de la Statistique (INS) [Niger], Unicef et ICF International 2012. *Enquęte Démographique et de Santé dans les Zones d’Intervention du Programme de Coopération de l’UNICEF au Niger, 2012*. Rockville, Maryland, USA: INS, Unicef et ICF International; and Institut National de la Statistique (INS) et Macro International Inc. Enquęte Démographique et de Santé et à Indicateurs Multiples du Niger 2006. Calverton, Maryland, USA: INS et Macro International Inc.; Février 2007. http://dhsprogram.com/pubs/pdf/FR193/FR193-NI06.pdf.

Further, between 2006 and 2012, the average annual rate of decline in under–five mortality was 6.6% in the intervention areas compared to 6% nationally (**Figure 3**). While this difference of 0.6% may appear to be small, the implications are considerable in a country such as Niger which had the highest fertility rate in the world in 2012 [19]. In addition, the average annual rate of decline in neonatal mortality was nearly twice as high in the programme intervention areas as the national decline over the same time period (**Figure 3**).

### Next steps and lessons learned

Available evidence suggests that these multi–faceted demand generation and social mobilisation activities contributed to improved utilisation of iCCM–related interventions in Niger. The findings from Niger’s experience suggest that community involvement and commitment are critical to reduce relevant bottlenecks in the access and use of services. Community *relais* and local leaders are key actors to ensure community engagement and empowerment. In addition, a better understanding of social norms can support efforts to reduce related bottlenecks relating to behaviours. Finally, efforts to strengthen local capacity in data analysis and use (for example, with user–friendly tools) can facilitate evidence–based programming.

Significant progress has been made towards reducing under–five mortality in Niger, although some challenges remain. Some of the most important demand–related barriers include lack of knowledge of danger signs, the competing household responsibilities of women, and continued confidence in the efficacy of traditional treatments, particularly in areas where there are no *relais* [25]. A recent case study examining Niger’s success in decreasing under 5 mortality indicates that the use of community–based providers to provide high–impact promotive, preventive and curative interventions at the peripheral health posts was a key factor in improving timely care–seeking for and life–saving treatment of childhood illnesses [26]. The Niger government and its partners are continuing to focus on addressing these important issues in order to support further achievements in child survival.

## MOZAMBIQUE EXPERIENCE: THE NATIONAL COMMUNITY HEALTH WORKER PROGRAMME AND COMMUNITY ENGAGEMENT

### Setting

Mozambique’s population reached 24.5 million people in 2012, and, similar to Niger, it has one of the lowest Human Development Index rankings in the world [18]. The under 5 mortality rate of 90 deaths per 1000 live births ranked 22nd highest in the world in 2012 [19], however this was a substantial decline from the rate of 219 per 1000 live births in 1997 [27]. The country is mostly rural (only 31 percent of the population lives in urban areas) and 60 percent of the population lives in poverty [19].

### Overview of the approach

The National Community Health Worker programme originally started in 1978 but was abandoned during the country’s protracted civil war. The programme was revitalized in 2010 and since early 2014 the programme has been under expansion with approximately 3800 CHWs (known in Mozambique as ‘*Agentes Polivalentes Elementares’* or *‘APEs’*) slated for training. Under Ministry of Health policy, these CHWs should each serve between 500 and 2000 inhabitants, though in practice, many serve more than 2000. CHWs should also live within the communities they serve, and provide a range of services encompassing health promotion for behaviour change, preventive home visits, screening for malnutrition and integrated community case management for childhood illnesses such as malaria, diarrhoea, and pneumonia. The goal of the CHW programme is to reduce gaps in access to preventive and curative care for remote communities by extending health services to an additional 20 percent of the population, and promoting actions for health and social change.

At the core of the CHW programme is a strong link with the Ministry of Health’s (MoH’s) comprehensive community engagement approach, established in the National Strategy for Health Promotion [28] and Terms of Reference for Establishing Community Health Committees (‘*Comités de Saúde Comunitário*’) [29], which focuses on community mobilisation and community participation in decision–making on health issues and in the selection of CHWs. The programme views community engagement as a continuum, starting with extensive community awareness raising, and community participation in selection of CHWs, and moving towards community mobilisation and involvement around critical health issues.

Additional strategies of the MoH’s community engagement approach are to build the capacity of both health personnel and partners in participatory methodologies, establish Co–management Committees which link facilities with communities, work in coordination with various community volunteers, local non–governmental organisations and community–based organisations, and to involve key community leaders (political, religious, traditional, teachers, and others) in decision–making and planning on health issues related to their communities.

Further, the programme works to ensure ‘effective access’ for services in order to increase care–seeking and treatment. According to the Mozambique Ministry of Health, in order to achieve ‘effective access’ the CHW must be equipped, accessible, available, motivated, and supervised.

### Programme methods and results

In 2009, the non–governmental organisations Malaria Consortium and Save the Children began supporting iCCM through the Ministry’s CHW programme in Inhambane and Nampula Provinces respectively. In addition to supporting “effective access” elements, including training, supervision, monitoring and provision of medicines for CHWs, attention was also given to community mobilisation for uptake of the new services provided by CHWs.

A national Symposium was held in Maputo in July 2013, co–hosted by the CHW (APE) Programme–Ministry of Health, Malaria Consortium and Save the Children, which brought together a wide range of stakeholders, including major donors, non–government organisations, UN agencies and local research groups, to present and discuss results and lessons learned from this 3–year programme and implications for community–based health programming in the country. In addition, the endline survey results were shared with donors, non–government organisations, research groups and other stakeholders at a meeting in the United States in May 2013, where findings and lessons learned from the three countries (Mozambique, South Sudan and Malawi) that received grants from the Government of Canada were presented.

**Figure 4:**
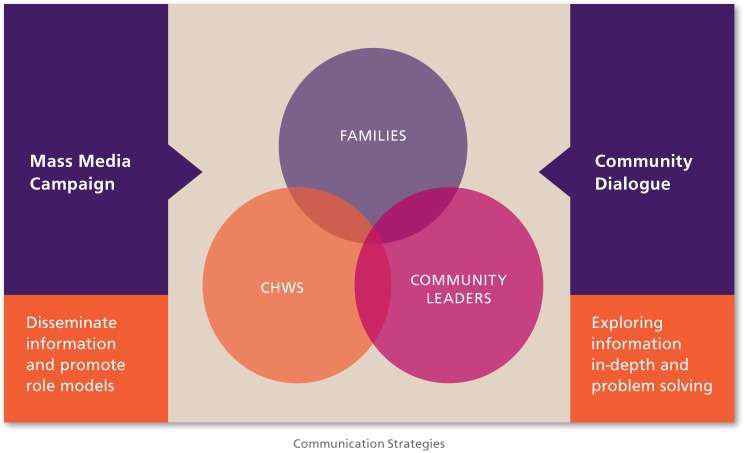
Overview of Mozambique’s community engagement strategy in the province of Inhambane. CHW – community health worker.

During the preparation phase in Inhambane Province, qualitative research studies were conducted to look at existing knowledge, attitudes and behaviours in relation to the prevention and management of diarrhoea, pneumonia and malaria in children. The results revealed some knowledge gaps and misconceptions among caregivers which contributed to inappropriate care–seeking and management practices. Most caregivers also showed awareness on a number of child health recommendations but low levels of self–efficacy in putting these recommendations in practice, especially in seeking medical treatment for their ill children. iCCM was generally highly acceptable to community members [30]. Findings also highlighted the lack of community support and involvement in the CHW programme at the time, reflecting low levels of community awareness and appreciation of the CHWs’ roles and responsibilities [31]. To address these issues a public health communication intervention was designed to improve health–care seeking and prevention practices around child health, through individual and collective actions. The intervention is based on a socio–ecological approach, which conceptualises individual behaviour as the result of overlapping individual, social and environmental issues [32]. It combines two main complementary strategies: the diffusion of information through mass media (namely radio programming) and community dialogues (**Figure 4**). The community dialogue (CD) model, which is based on participatory learning and action approaches, was integrated in the existing iCCM programme in Inhambane province to strengthen the CHWs’ health promotion activities. Intensive community sensitisation efforts were conducted prior to the 2011 deployment of 292 CHWs in Inhambane Province, which was followed about a year later by the introduction of the CD intervention. For each community (defined as the CHW’s catchment area), the CHW and his or her corresponding community leader were trained on a simple 10–step methodology and provided with visual tools (available at www.ccmcentral.org) that allowed them to engage local communities in monthly participatory discussions around prevention and optimal management of childhood diseases through iCCM services and in collectively identifying barriers and solutions. To position CHWs’ services in the public sphere and reinforce their community–level promotional activities, a daily radio edutainment programme was broadcasted in prime time from August to November 2012. The approach addresses a set of constructs, such as knowledge of disease and danger signs as well as services available, self–efficacy and social norms, including that CHWs can be a first choice for care.

A qualitative process evaluation of the intervention was conducted in 2013 using a method described by Saunders et al [33]. The evaluation consisted of 29 focus groups and 38 key informant interviews, complemented with secondary monitoring data, including structured CD observations, CD monitoring sheets and programme reports [34]. The evaluation found an increased awareness and appreciation of the CHWs by the community members, who consider them to be ‘health leaders.’ The model also seems to have contributed to filling some knowledge gaps. Community members interviewed considered CDs as a major source of information and demonstrated correct knowledge of the causes and prevention measures for diarrhoea and malaria, but pneumonia appeared to be rarely discussed in CDs. Among communities visited, people who had attended CDs considered CHWs as their first choice for care and indicated that using CHWs had become a habit. The findings also highlighted the importance of the local leadership: its presence and involvement in the activities legitimized the role of the CHWs and gave more weight to the messages of health promotion and prevention conveyed by the CHWs. The evaluation indicates that CD is an effective tool for setting new social norms and moving from information to action through the commitments agreed upon in public. As an example, through CDs, one community agreed with traditional medicine practitioners that all sick young children and people presenting with cough should be redirected to a CHW.

In order to measure changes in care seeking behaviour, morbidity and access to appropriate treatment (either from a CHW or a health facility) for children under five sick with malaria, pneumonia or diarrhoea, an outcome evaluation of the CHW programme in the province of Inhambane was conducted in collaboration with the Provincial Health Directorate. The evaluation involved a baseline household survey, in 2010, just before the beginning of programme implementation, and an endline survey in 2012. A two–stage sampling design was used and data were collected from a sample of 1409 households at baseline [35] and from a sample of 3032 households at endline [36]. All rural districts of the province were included in the sampling frame.

**Figure 5:**
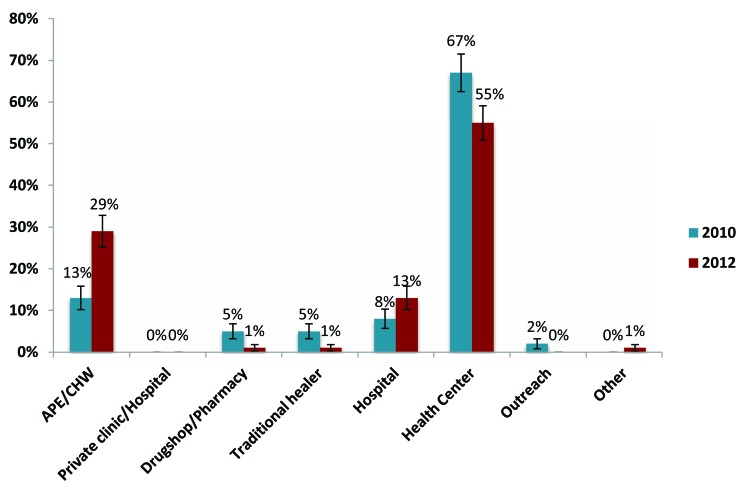
Primary source of treatment when any assistance was sought, Inhambane Province Mozambique, 2010 and 2012.

Results indicated a trend of significant progress in both diagnosis and treatment seeking behaviours as a result of the presence of the CHWs in the communities. As shown in **Figure 5**, when care was sought for childhood illness, treatment provided by CHWs increased nearly 3–fold between 2010 and 2012, from 13% (95% confidence interval (CI) 10.2–15.8) to 29% (95% CI 25.2–32.8). While the public sector remained the primary source of care at endline, the use of private sector sources (including drug shops/ pharmacies and traditional healers) decreased in favour of the CHWs.

**Figure 6:**
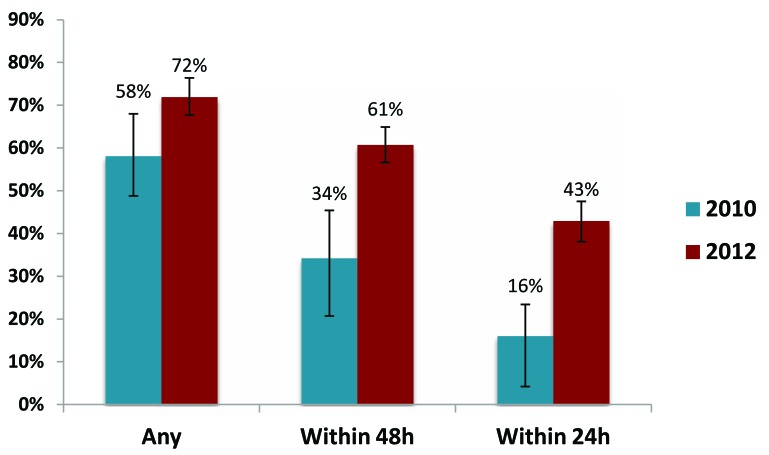
Care–seeking for childhood illnesses, overall and by timeliness, Inhambane Province Mozambique, 2010 and 2012.

**Figure 7:**
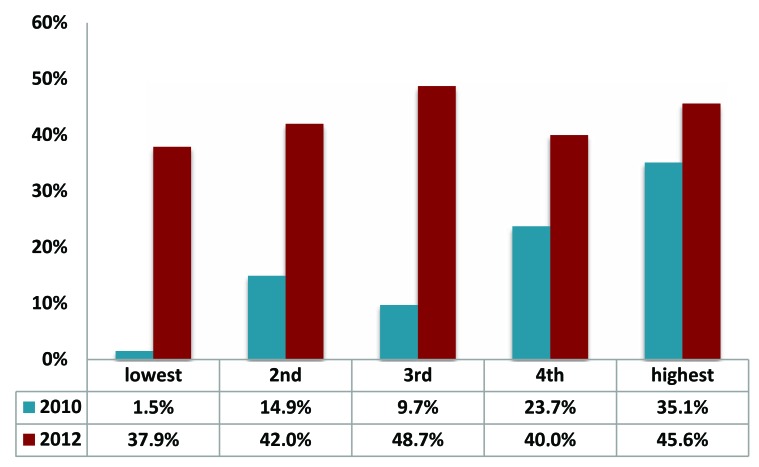
Care–seeking for childhood illnesses within 24 hours by wealth quintile, Inhambane Province Mozambique, 2010 and 2012.

Furthermore, the deployment of CHWs had a positive effect on timeliness of care–seeking in the province: treatment seeking within the first 24 hours of symptom onset increased from 16% (95% CI: 8.6–27.8) in 2010 to 42.9% (95% CI: 38.3–47.7) in 2012 (**Figure 6**). As shown in **Figure 7**, this effect was particularly important for families living within the poorest quintiles. The equity ratio for treatment within 24 hours increased from 0.04 to 0.83 over the two year period so that this behaviour changed from being virtually non–existent among the poorest families to being almost as common as among the richest families. The survey also found that, among caretakers who had not mentioned the CHW as their primary source of treatment at endline survey, more than two thirds either did not know the CHW in their community (44.7%; 95% CI 41.5–47.9) or preferred another source (23.9%; 95% CI 21.1–26.7). The other reasons for not using the CHW services were distance (11.5%; 95% CI 9.4–13.6), lack of medicines (10.6%; 95% CI 8.6–12.6) and non–availability (7.2%; 95% CI 5.5–8.9).

This indicates that a number of households are still not being reached regularly by community mobilisation interventions in a context where CHW catchment areas can be very large, and that the proportion of all ill children primarily seen by a CHW should further increase with better recognition of the CHW within the community and better supply of the CHWs with medicines. More efforts and investments need to be put in place to better understand barriers and expand service outreach, including through the training and deployment of additional CHWs combined with the continuation and intensification of community mobilisation activities to leverage greater results for child survival.

In 2010, Save the Children trained and deployed 291 CHWs in Nampula province. From August 2010 until December 2012, Save the Children provided supervision support to CHWs and key iCCM medicines (amoxicillin, ACTs and RDTs) to supplement the CHW medical kit. To support the MoH at district level, Save the Children contracted district supervisors to provide intensive supervision to CHWs during the initial stages of the program. At community level, Save the Children worked together with CHWs and community leaders to promote community engagement in the CHW programme.

In order to promote acceptability and uptake of iCCM services, in February and August 2012, 1330 Community Health Committee (CHC) members were trained on key community–related components of iCCM, including recognition of danger signs and prevention of the three iCCM illnesses targeted by the CHW program. In collaboration with the district health offices, Save the Children created a flipchart on identifying danger signs and corresponding actions for prompt and appropriate care–seeking. This flip chart was used as tool during the training and a ‘job aid’ for community health committee members after the training. The training package, which focused primarily on iCCM, was developed before the MOH’s national comprehensive community engagement strategy was finalised. In February and August 2012, Save the Children also partnered with community radio stations to design, test and broadcast radio messages to raise communities’ awareness on the iCCM services provided by the CHWs, the importance of early care–seeking, and to encourage communities to adopt appropriate preventive practices. Messages were broadcast twice weekly. Themes and content of messages were based on results of the project's baseline survey, conducted in 2010, and targeted specific areas where caregivers had limited knowledge related to prevention and care–seeking for the three iCCM illnesses.

In collaboration with the Nampula Provincial Health Department the project was evaluated in October 2012 in order to assess the effects of the iCCM programme on care seeking for childhood malaria, pneumonia and diarrhoea and appropriateness of treatment. The survey used a three–stage cluster sampling design stratified by intervention and comparison areas (600 households with children under five were sampled in each arm). The CHW catchment areas served as clusters in intervention areas while census enumeration areas (primary sampling units) eligible for iCCM served as clusters in comparison areas.

**Figure 8:**
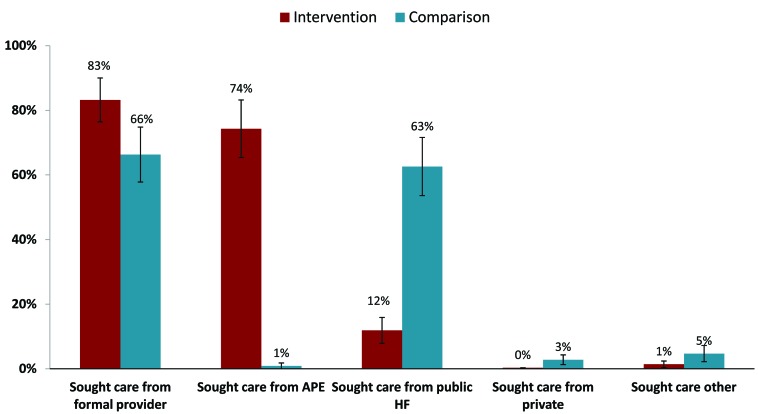
Care–seeking for fever among children under 5 years in intervention and comparison areas in Nampula Province Mozambique, 2012. APE – *Agentes Polivalentes Elementares*, HF – health facility.

**Figure 8** shows that demand for iCCM services in the form of care seeking for fever from a formal provider was significantly higher in the Nampula intervention clusters (83.2%; 95% CI 76.3–90.0) than comparison areas (66.3%; 95% CI 57.8–74.9). In addition, CHWs were the main source of care seeking in intervention areas with nearly three–quarters (74.3%; 95% CI 65.4–83.2) of all children with fever being taken to a CHW, accounting for 89.3% of total care seeking from public sector providers (269/301).

For each intervention cluster, a variable was created using data from a complementary survey of 30 CHWs. The variable indicated whether the CHW serving that cluster was active (residing in catchment area and a register review indicated that sick child cases were treated in the past 7 days) or inactive (not residing in catchment area and/or had not treated sick child cases in the past 7 days). Of the 30 intervention clusters, 24 had a CHW who met the criteria for active. Results showed that active care–seeking for fever was 34 percentage points higher among active CHWs (82% vs 48%).

**Figure 9:**
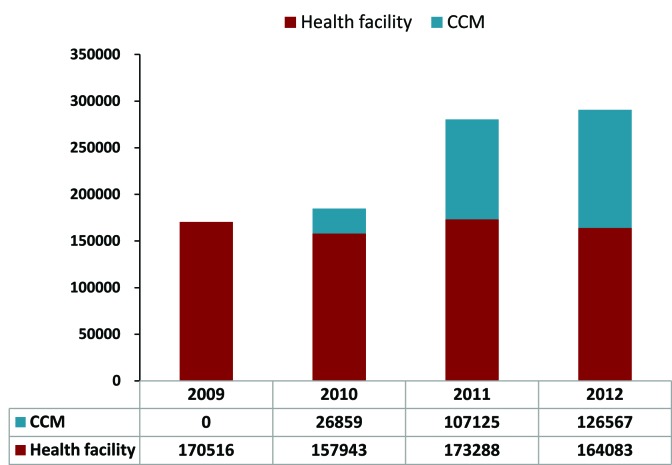
Malaria treatment by source in 10 iCCM districts in Nampula Province, Mozambique, 2012 to 2012. CCM – community case management.

Analysis of routine monitoring data from CHWs and health facilities over the time period from 2009 (when iCCM services for malaria were largely unavailable) to 2012 shows that demand from CHWs increased. By 2012, CHWs were treating 44% (126 567/290 650) of all malaria cases in the 10 districts of Nampula Province (**Figure 9**). At the same time, the number of cases treated at facilities remained relatively stable and did not show a pattern of demand replacement, ranging from 170 516 malaria treatments at facilities in 2009 to 164 083 in 2012.

### Next steps and lessons learned

Mozambique’s experience shows that a comprehensive community engagement and empowerment strategy focused on improving health outcomes of young children, along with ensuring effective access to CHWs can result in positive results for families with young children, particularly those living in remote settings of the country with poor access to health services. In this experience, community readiness and support from local leadership was a key for accountability and social mobilisation, as was collaboration and coordination between CHWs and Community Health Committees. Mobilisation efforts utilised interpersonal dialogue with community members (ie, the teams went beyond simple ‘messaging’) and as a result, built trust, cooperation and programme ownership. Further, iCCM activities proved to be more effective because they were integrated into a comprehensive approach including health promotion and prevention, and into a larger community involvement programme. Finally, the Mozambique experience revealed that it was critical to ensure that CHWs have a regular supply of medicines and equipment in order to maintain demand once it was generated.

There are important issues to consider as the programme continues to expand throughout the country, including how best to respond to recurrent demands from community members for health posts in their communities, expansion of CHW roles to include additional tasks, and for better quality of care at health facilities.

## DISCUSSION

This analysis is subject to several limitations, including the fact that a case study approach was utilised, which is not generalizable and does not lend itself towards numerical representation.

The evaluations conducted in Niger and Mozambique assessed the overall impact of the iCCM programme in terms of increased access to appropriate treatment of sick children, but could not identify the respective contribution of each programme component separately. In Niger, although some process data were collected, documentation of these activities was limited. Further, data were not weighted in the specific survey conducted in Niger to assess outcomes based on the KFP intervention. Using weighted data might have revealed additional (or different) findings for intervention vs control areas. However the availability of DHS data in 2012 provides another measure to validate the positive findings of the KFP survey.

In Mozambique, the endline and baseline surveys did not measure exposure to communication and community mobilisation interventions thus not allowing for dose–response efficacy analysis. The evaluation also suffered from the lack of agreed upon indicators and methods to measure the specific outcomes of community engagement activities. With the exception of the Community Dialogue intervention in Inhambane province for which Malaria Consortium developed specific monitoring and evaluation tools and sourced additional funding to evaluate it, process data on community mobilisation activities are scarce. Despite community engagement being a key component of the CHW programme in Mozambique, the national monitoring and evaluation system and tools do not capture process data which could further be analysed to monitor demand level and barriers and inform further programme improvements.

Still, the experiences of these two diverse settings may hold relevance to other resource–poor settings with a need to generate demand for life–saving child health interventions. In addition, the teams implementing the projects conducted rigorous analyses to demonstrate outcomes in the intervention and control areas.

Based on the experiences of these two case studies of iCCM and child survival demand generation and social mobilisation activities, several strategies appear to have worked well.

First, demand increased, both for iCCM services and as well as other child health priorities, following the implementation of comprehensive social mobilisation efforts. These efforts incorporated interpersonal communication activities and community empowerment/participation for collective change, partnerships and networks among key stakeholder groups within communities, media campaigns and advocacy efforts with local and national leaders. In these settings, social mobilisation and community participation improved community ownership (something that may lead to improved programme sustainability) [37,38], and helped the community interact with and support the health system (for example, to discuss bottlenecks in access and quality of services, to identify locally relevant solutions, and to improve the flow of information across partners).

Second, these efforts involved a participatory process for community selection of local individuals to work as CHWs, something that can facilitate community acceptability of CHW services and for the CHWs themselves [39,40].

Third, community members were made aware of the skills and training of CHWs in order to build trust in the services they provide. Caregivers seek care from providers whose services they trust and respect, and who show respect for them [41–43]. In settings where caregivers have a variety of provider options (including where other provider types such as traditional healers and drug shops are well established), it can be particularly important that families understand who the CHWs are and what they offer.

Fourth, both approaches described here incorporated efforts to make community members aware of danger signs and appropriate treatments for illnesses, key factors in improving prompt and appropriate care–seeking [44–46].

Fifth, in these settings, CHWs were allowed to treat for more than one disease, something that may generate higher demand as families often seek care for more than one problem. One study in Uganda reported poor caregiver compliance to referral to facilities, as well as a negative impact on families’ confidence in the community programme, when only malaria (and not pneumonia) treatment was offered in the community [47].

Finally, these approaches incorporated efforts to ensure availability of supply side elements that can influence demand. They worked to ensure local availability and appropriate density of CHWs as well as a consistent and high quality supply of medicines, both of which have been found to correlate to improved care–seeking and utilisation for iCCM services in other settings [13,15,17,48–51]. One Ugandan study reported that following a stock out in a CCM program, caregivers continued to bypass CHWs even after the drug supply problem was rectified [51].

However, as Ensor and Cooper have noted [4], it is critical to address demand issues through demand side–specific interventions, not just as an adjunct to implementation but rather as a primary component within programme packages. Community mobilisation has been recognised as one of the key features for successful health interventions, and the literature confirms [52] that interventions designed to maximize community collaboration and participation can have a beneficial impact on child health. However evidence is still scarce on what does or does not work [53] and further research is needed which should pay specific attention to the collection of information on the continuum of community approaches and carefully evaluating the implementation processes [52].

Additional research is needed in order to understand how to maximize appropriate demand for iCCM services. Operational research to test and compare the respective impact of specific approaches would bring valuable insights to programme managers and help develop clear rationale for the selection of the most appropriate approach to each context. Other areas of interest include improving our understanding of the role of CHW gender in acceptability of iCCM services (which will be of particular importance as iCCM services expand to include maternal and neonatal treatments), how to improve caregiver understanding of the differences between simple cough and cold and pneumonia (in order to reduce demands for unnecessary antibiotic treatment) and how to use existing data sources to capture local demand for and barriers to iCCM services. Further, more information is needed on how best to promote adherence to treatment. In Uganda and Zambia, incorporating RDTs into iCCM programmes was found to have a positive impact [45,54].

## CONCLUSION

iCCM programmes, when implemented with careful attention to training CHWs, ensuring adequate drug supplies and mobilizing and engaging community members and stakeholders to access and participate in services, cannot only increase care–seeking among families with sick children, but they can also improve the timeliness and appropriateness of care–seeking. iCCM programmes can also replace facility–based care (thereby reducing facility caseloads) and care from other sources such as drug shops and traditional healers, improving timeliness and in some cases appropriateness of treatment.

Generating demand is not simple. The barriers to seeking appropriate treatment are complex and are affected by myriad factors, both financial and non–financial. As a result, it may not always be possible to see quick changes in care–seeking behaviors once iCCM services are made available. But the experiences described above show that iCCM programmes can positively influence demand for and uptake of treatment services, provided that CHWs are trained and equipped with interpersonal communication tools and methods and supported by wider community engagement approaches.
